# The Combination of Structure Prediction and Experiment for the Exploration of Alkali‐Earth Metal‐Contained Chalcopyrite‐Like IR Nonlinear Optical Material

**DOI:** 10.1002/advs.202106120

**Published:** 2022-04-11

**Authors:** Peng Wang, Yu Chu, Abudukadi Tudi, Congwei Xie, Zhihua Yang, Shilie Pan, Junjie Li

**Affiliations:** ^1^ CAS Key Laboratory of Functional Materials and Devices for Special Environments Xinjiang Technical Institute of Physics & Chemistry CAS Xinjiang Key Laboratory of Electronic Information Materials and Devices Urumqi 830011 P. R. China; ^2^ Center of Materials Science and Optoelectronics Engineering University of Chinese Academy of Sciences Beijing 100049 P. R. China; ^3^ Skolkovo Institute of Science and Technology Skolkovo Innovation Center 3 Nobel Street Moscow 143026 Russian Federation

**Keywords:** alkaline earth metals, chalcogenide, chalcopyrite‐like structures, nonlinear optical materials, tetrahedral units

## Abstract

Design and fabrication of new infrared (IR) nonlinear optical (NLO) materials with balanced properties are urgently needed since commercial chalcopyrite‐like (CL) NLO crystals are suffering from their intrinsic drawbacks. Herein, the first defect‐CL (DCL) alkali‐earth metal (AEM) selenide IR NLO material, DCL‐MgGa_2_Se_4_, has been rationally designed and fabricated by a structure prediction and experiment combined strategy. The introduction of AEM tetrahedral unit MgSe_4_ effectively widens the band gap of DCL compounds. The title compound exhibits a wide band gap of 2.96 eV, resulting in a high laser induced damage threshold (LIDT) of ≈3.0 × AgGaS_2_ (AGS). Furthermore, the compound shows a suitable second harmonic generation (SHG) response (≈0.9 × AGS) with a type‐I phase‐matching (PM) behavior and a wide IR transparent range. The results indicate that DCL‐MgGa_2_Se_4_ is a promising mid‐to‐far IR NLO material and give some insights into the design of new CL compound with outstanding IR NLO properties based on the AEM tetrahedra and the structure predication and experiment combined strategy.

## Graphical abstract

The first defect‐chalcopyrite‐like alkali‐earth metal selenide IR NLO material DCL‐MgGa_2_Se_4_ with balanced SHG response and band gap is rationally designed and fabricated by a calculation and experiment combined strategy.

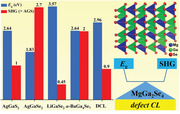

## 1 Introduction

NLO materials play an important role in modern laser science and technology. Based on frequency conversion technology, NLO crystals can expand the spectral range of laser sources, which are widely applied in laser photolithography, long distance laser communication, environmental monitoring, and photonic technologies.^[^ ^1^ ^]^ In IR regions, CL AgGaS_2_, AgGaSe_2_ (AGSe), and ZnGeP_2_ (ZGP), showing large second harmonic generation (SHG) responses, are the commonly commercial IR NLO materials.^[^ ^2^ ^]^ Nevertheless, owing to the intrinsic drawbacks like small band gaps in these materials, which induce low laser induced damage threshold (LIDT) in AGS and AGSe, non‐PM behavior in AGSe, and unexpected two‐photon absorption (TPA) in ZGP, the application of these materials are limited in current laser techniques, especially for the output of high‐powder laser.^[^ ^3^ ^]^ Therefore, it is an urgent need to design and fabricate new IR NLO materials with wide band gap (preferably >2.33 eV to avoid TPA of 1064 nm laser and to achieve high LIDT), large SHG coefficients (preferably >10 pm·V^−1^, comparable to AGS), wide mid‐to‐far IR transparent regions that covering the 3–5 and 8–12 µm windows, moderate birefringence (>0.04) to achieve type‐I PM behavior, as well as good crystal growth habits,^[^ ^4^ ^]^ but challenging because of the potential conflicts between wide band gap and large SHG response, birefringence, as well as IR transparent range.^[^ ^5^ ^]^

Chalcogenides, with abundant structural diversity, have been widely used for the exploration of IR NLO materials.^[^ ^6^ ^]^ Among them, selenides normally exhibit wider IR transparent regions, larger SHG responses, and lower melting points, but smaller band gaps than sulfides.^[^ ^7^ ^]^ Over the past decades, a plenty of selenides with good NLO properties like LiGaSe_2_,^[^ ^8^ ^]^ *α*‐BaGa_4_Se_7_,^[^ ^9^ ^]^ *β*‐BaGa_4_Se_7_,^[^ ^10^ ^]^ *β*‐BaGa_2_Se_4_,^[^ ^11^ ^]^ Li_2_ZnMSe_4_ (M = Ge, Sn),^[^ ^12^ ^]^ Li_2_CdMSe_4_ (M = Ge, Sn),^[^ ^13^ ^]^ Li_2_In_2_MSe_6_ (M = Si, Ge),^[^ ^14^ ^]^ Na_4_MgM_2_Se_6_ (M = Si, Ge),^[^ ^15^ ^]^ BaGa_2_MSe_6_ (M = Si, Ge),^[^ ^16^ ^]^ *γ*‐NaAsSe_2_,^[^ ^17^ ^]^ Na_6_MSe_4_ (M = Zn, Cd),^[^ ^18^ ^]^ CsM_3_Se_6_ (M = Ga/Sn, In/Sn),^[^ ^19^ ^]^ and AgLiGa_2_Se_4_^[^ ^20^ ^]^ have been designed and synthesized successfully. However, to enhance the LIDT, the band gaps in selenides are still highly expected to be further improved. For the design of new IR NLO materials, atomic substitution has been demonstrated as a feasible strategy, and many new compounds have been developed in this way by numerous experimental attempts.^[^ ^21^ ^]^ However, the trial‐and‐error processes are time‐consuming, and plenty of attempts are not always effective.^[^ ^22^ ^]^ Recently, computer‐aided material fabrication, based on crystal structure prediction, property calculation, and screening techniques, has become an effective way for exploring targeted functional materials like superconducting materials, electrode materials in battery, and ultraviolet NLO materials.^[^ ^23^ ^]^ Nevertheless, the successful case for chalcogenide IR NLO materials is scare.^[^ ^24^ ^]^

In this work, a new IR NLO material DCL‐MgGa_2_Se_4_ has been designed and fabricated by the calculation and experiment combined strategy. For targeted design, non‐centrosymmetric (NCS) DCL AB_2_X_4_ metal‐chalcogenide was utilized as the initial structural template, and AEM tetrahedral units, NLO‐active GaSe_4_ were tried to introduce into the template to balance the band gap and SHG response. In view of the replaceability of tetrahedral units in CL structure and the relative high formation probability of MgSe_4_, we believe that it is possible to explore AEM CL IR NLO materials with balanced properties in Mg–Ga–Se system. The theoretical predictions and experimental investigations demonstrate the existence of DCL‐MgGa_2_Se_4_ in AB_2_X_4_ family. To the best of our knowledge, DCL‐MgGa_2_Se_4_ is the first ternary DCL AEM selenide on basis of the statistical analysis in Inorganic Crystal Structure Database (ICSD ‐ 4.7.0, the latest release of ICSD ‐ 2021/10/25) (Table S1, Supporting Information). The compound crystallizes in tetragonal *I* $\bar{4}$ (No. 82) space group with unit cell *a* = 5.6997(2) Å, *c* = 10.7265(6) Å, *Z* = 2. The compound exhibits a balanced optical properties including a wide band gap of 2.96 eV, suitable SHG response (≈0.9 × AGS) with PM behavior, and large LIDT (≈3.0 × AGS).

## 2 Results and Discussion

**Figure 1:**
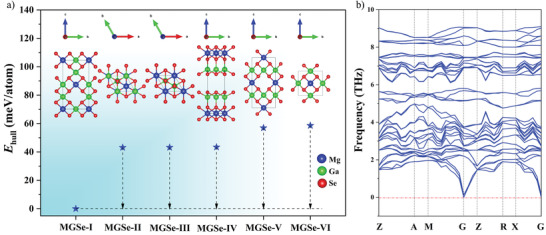
a) Crystal structures and the corresponding *E*_hull_ of each phase of MGSe (I–VI) and b) phonon dispersion spectrum of MGSe‐I.

CL compounds are still attractive structural templates for the design of new IR NLO materials due to their successes in AGS, AGSe, and ZGP. To increase their band gap, introducing AEM tetrahedral units without *d*–*d* and *f*–*f* electron transition into the CL compounds is highly expected.^[^ ^25^ ^]^ However, owing to the strong ionicity between AEM and chalcogenide atoms, AEMs tend to form MSe*_n_* (*n* ≥ 6) polyhedral groups rather than MSe_4_ tetrahedra. Hence, AEMs were seldom used for the design and fabrication of CL IR NLO materials in previous investigations. Based on statistical analyses on all the reported AEM‐contained selenides in ICSD, BeSe_4_ and MgSe_4_ units can be found in some known compounds, like in sphalerite‐like BeSe,^[^ ^26^ ^]^ wurtzite‐like MgSe,^[^ ^27^ ^]^ and spinel‐like MgTm_2_Se_4_,^[^ ^28^ ^]^ MgYb_2_Se_4_^[^ ^28^ ^]^ with short Be‐Se (2.119 Å) and Mg‐Se (2.542 ‐ 2.643 Å) bonding distances, while Ca, Sr, and Ba atoms tend to form MSe*_n_* polyhedral groups with long Ca—Se (2.856–3.225 Å), Sr—Se (2.944–3.569 Å), and Ba—Se (3.106–3.865 Å) bonding distances (Figure S1, Supporting Information). Moreover, MgSe_4_ exhibits a high formation probability of ≈50% in the Mg‐contained selenides without cationic co‐occupation (Figure S1, Supporting Information). Therefore, grouping the rigid MgSe_4_ (Be is toxic) with NLO‐active GaSe_4_ tetrahedral units into CL structure is theoretically feasible for the design and exploration of new IR NLO materials. To verify this point, the computer‐aided structure predictions were performed in the ternary Mg–Ga–Se system by the ab initio evolutionary algorithm USPEX (Universal Structure Predictor: Evolutionary Xtallography) structure prediction method.^[^ ^29^ ^]^ The structures of MgGa_2_Se_4_ (MGSe) I–VI were predicted by using global evolutionary algorithm at 0 K and at standard atmospheric pressure (**Figure** **1**a and Table S2, Supporting Information). Their *E*_hull_ (the computed formation enthalpies above thermodynamical convex hull) were calculated based on MgSe and Ga_2_Se_3_. The DCL MGSe‐I (*I* $\bar{4}$, *Z* = 2 ) shows the lowest formation enthalpy. Compared with MGSe‐I phase, the predicted MGSe‐II (*R* $\bar{3}$ *m*, *Z* = 3), MGSe‐III (*P* $\bar{3}$ *m*1, *Z* = 1), MGSe‐IV (*C*2*/m*, *Z* = 2), MGSe‐V (*I* $\bar{4}$2*m*, Z = 2), and MGSe‐VI (*P* $\bar{4}$2*m, Z* = 1) exhibit higher formation enthalpies. It's worth noting that the predicted centrosymmetric phase MGSe‐II with high *E*_hull_ is also thermodynamic stable, which has been reported by Kim et al. in 1988,^[^ ^30^ ^]^ while the NCS MGSe‐I phase with low *E*_hull_ has not been synthesized. To evaluate the thermodynamic stability, the phonon spectrum of MGSe‐I was investigated. Phonon calculations verify that MGSe‐I is dynamically stable owing to the absence of an imaginary phonon mode from the Brillouin zone (Figure 1b). The results indicate that there is an unexplored ground state MGSe‐I phase in the ternary Mg–Ga–Se system. Furthermore, the calculated PBE0 band gaps of MGSe (I–VI) are 3.101, 1.548, 1.577, 2.371, and 2.454 eV, respectively, confirming the crystal structures affected optical properties and the large band gap in the NCS DCL MGSe‐I phase. What's more, the calculated NLO coefficients of MGSe‐I are 19.88 pm·V^−1^ for *d*_14_ and 3.33 pm·V^−1^ for *d*_15_. The calculated results indicate the ground state MGSe‐I phase could be a potential IR NLO material with balanced band gap and SHG response.

Different from previous structural predictions,^[^ ^31^ ^]^ one of the significant breakthroughs is that the predicted DCL‐MgGa_2_Se_4_ (MGSe‐I phase) was fabricated by high‐temperature solid state reactions, and the purity of the synthesized samples was verified by the powder X‐ray diffraction (PXRD) (Figure S2, Supporting Information). The crystal structure was resolved by single‐crystal X‐ray diffraction (SXRD), which is consistent with the predicted results. The bond valence sum (BVS)^[^ ^32^ ^]^ calculations (Mg = 2.063, Ga = 2.947–2.995, Se = 2.001) indicate that all atoms are in reasonable oxidation states. **Table** **1** shows the relevant crystallographic data and structural refinement details. To further confirm the chemical compositions, the energy dispersive X‐ray spectroscopy (EDS) spectrum on DCL‐MgGa_2_Se_4_ single crystal was characterized. The results confirm the existence of Mg, Ga, and Se elements, and the atomic ratio is quantified to Mg:Ga:Se = 13.51:27.98:58.50 in the compound (Figure S3, Supporting Information), which matches with the ratio of 1:2:4 in the formula of DCL‐MgGa_2_Se_4_.

**Table 1:** Crystal data and structural refinement for DCL‐MgGa_2_Se_4_

Chemical formula	MgGa_2_Se_4_
Formula weight	479.59
Crystal system	Tetragonal
Space group	*I* $\bar{4}$
*a* [Å]	5.6997(2)
*c* [Å]	10.7265(6)
Volume [Å^3^]	348.47(3)
*Z*, *ρ*_calcd_ [Mg·m^−3^]	2, 4.571
μ [mm^−1^]	28.585
*F*(000)	420
Theta range for data collection [°]	3.799 to 27.496
Reflections collected/unique	2687/405 [*R*_int_ = 0.0681]
Completeness [%]	98.2
Data/restraints/parameters	405/0/17
GOF on *F^2^*	0.939
Final *R* indices [*I* > 2sigma(*I*)]^a)^	*R*_1_ = 0.0194, *wR*_2_ = 0.0401
*R* indices (all data)^a)^	*R*_1_ = 0.0194, *wR*_2_ = 0.0402
Absolute structure parameter	0.04(4)
Largest diff. peak, hole (e·Å^−3^)	0.535, −0.671

^a)^ *R*(*F*) = Σ||*F*_o_| – |*F*_c_||/Σ|*F*_o_|, and *wR*(*F*_o_^2^) = [Σ*w*(*F*_o_^2^ – *F*_c_^2^)^2^/Σ*wF*_o_^4^]^1/2^ for *F*_o_^2^ > 2*σ*(*F*_o_^2^).

**Figure 2:**
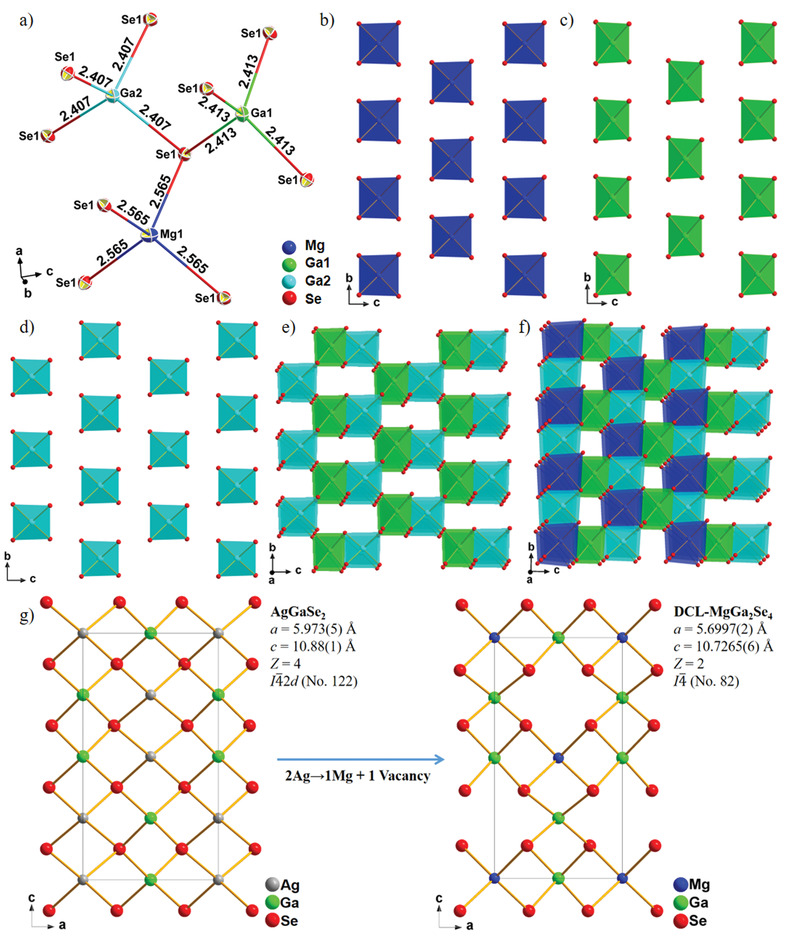
a) Asymmetric unit in DCL‐MgGa_2_Se_4_; b) isolated [MgSe_4_] tetrahedra in *bc* plane; c) [Ga1Se_4_] tetrahedra in *bc* plane; d) [Ga2Se_4_] tetrahedra in *bc* plane; e) [Ga_2_Se_7_]^8−^ anionic framework constructed by corner‐sharing [Ga1Se_4_] and [Ga2Se_4_]; f) the 3D structure of DCL‐MgGa_2_Se_4_; g) Structural evolution from AGSe (left) to DCL‐MgGa_2_Se_4_ (right).

DCL‐MgGa_2_Se_4_ is a uniaxial DCL compound and crystallizes in the NCS tetragonal *I* $\overset{-}{4}$ (No. 82) space group with *a* = 5.6997(2) Å, *c* = 10.7265(6) Å, and *Z* = 2 (Tables S1 and S3–S6, Supporting Information). In its asymmetric unit, there are one crystallographically unique Mg atom, two Ga atoms, and one Se atom (**Figure** **2**a). The Mg and Ga atoms are tetrahedrally coordinated with Se atoms to build [MgSe_4_] and [GaSe_4_] units (Figure 2a). The bond lengths of Mg—Se and Ga—Se are 2.565 Å in [MgSe_4_], 2.407 Å in [Ga1Se_4_] and 2.413 Å in [Ga2Se_4_], which are all within the rational bond length ranges.^[^ ^11^, ^33^ ^]^ The formed [MgSe_4_] tetrahedral units are isolated with each other (Figure 2b), similar to [Ga1Se_4_] (Figure 2c) and [Ga2Se_4_] units (Figure 2d). [Ga1Se_4_] units connect with adjacent [Ga2Se_4_] units by corner‐sharing to form [Ga_2_Se_7_] dimers, which are further connected with each other to build a [Ga_2_Se_7_]^8−^ anionic framework (Figure 2e) with rectangle pseudo‐channels viewed along *a* direction. The [MgSe_4_] tetrahedral units are located in the pseudo‐channels to construct the DCL 3D structure of the compound (Figure 2f). The crystal structure of DCL‐MgGa_2_Se_4_ can be derived from the chalcopyrite IR NLO material AGSe, as shown in Figure 2g.^[^ ^34^ ^]^ When two Ag atoms in AGSe are replaced by one Mg atom and one vacancy, DCL‐MgGa_2_Se_4_ is formed. Moreover, as we mentioned above, the MGSe‐II phase crystallized in centrosymmetric (CS) $R\overset{-}{3}m$(No. 166) space group with the cell parameters of *a* = 3.95 Å, *c* = 38.893 Å, and *Z* = 3.^[^ ^30^ ^]^ In the structure, Mg atoms are coordinated with six Se atoms to build MgSe_6_, and Ga atoms are coordinated with four Se atoms to build GaSe_4_. MGSe‐II shows a ZnIn_2_S_4_‐type structure constructed by the MgSe_6_ and GaSe_4_ groups, which is different from the DCL structure in MGSe‐I (DCL‐MgGa_2_Se_4_).

**Figure 3:**
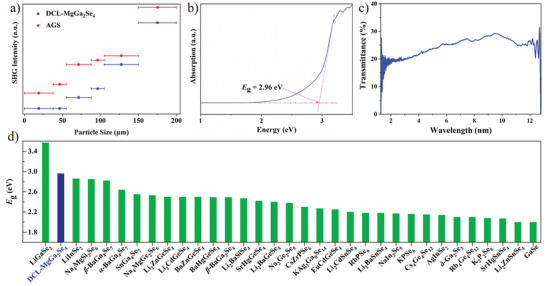
a) SHG intensity versus particle sizes with AGS as the references at 2.09 µm radiation, b) experimental band gap, and c) optical transmittance spectrum (measured by using single crystal) of DCL‐MgGa_2_Se_4_. d) The experimentally verified PM selenide IR NLO materials (without cationic co‐occupation) with *E*_g_ ≥ 2.0 eV.

The SHG responses of DCL‐MgGa_2_Se_4_ powder samples were evaluated by the Kurtz–Perry method with a 2.09 µm Q‐switch laser, and AGS samples were used as the references.^[^ ^35^ ^]^ As shown in **Figure** **3**a, the compound shows a type‐I PM behavior with a SHG response of ≈0.9 × AGS. Moreover, compared to the typical IR NLO materials like AGS, AGSe, LiGaSe_2_, and *α*‐BaGa_4_Se_7_, DCL‐MgGa_2_Se_4_ shows a good balance between strong SHG response and wide band gap (Figure S4, Supporting Information). The experimental optical band gap of DCL‐MgGa_2_Se_4_ is measured to be ≈2.96 eV through UV–Vis–NIR diffuse reflectance spectrum using the Kubelka–Munk function (Figure 3b).^[^ ^36^ ^]^ Based on the statistical analyses in Figure 3d and Table S7, Supporting Information, the band gap of the title compound is wider than most of the selenide‐based IR NLO materials, which is sufficient to eliminate the harmful effect of TPA under the 1064 nm pumping, and more prominent than those of benchmark IR NLO materials (AGS: 2.64 eV; AGSe: 1.83 eV; ZGP: 2.0 eV; *α*‐BaGa_4_Se_7_: 2.64 eV),^[^ ^2^, ^9^ ^]^ as well as the series of AB_2_X_4_ CL compounds like ZnGa_2_Se_4_^[^ ^37^ ^]^ (2.47 eV), CdGa_2_Se_4_^[^ ^37^ ^]^ (2.47 eV), and HgGa_2_Se_4_^[^ ^37^ ^]^ (1.80 eV) (Figure S5, Supporting Information). The large band gap inherently contributes to a large LIDT in DCL‐MgGa_2_Se_4_. The LIDT of the title compound was evaluated by single‐pulse LIDT method using a 1.06 µm incident laser (10 ns, 10 Hz). Under the same measurement conditions, the LIDT of DCL‐MgGa_2_Se_4_ is ≈3.0 times that of AGS. The optical transparent property of the compound was evaluated in the Fourier transformer infrared system (Bruker Vertex 80v) equipped with a Hyperion microscope by using the micro‐scale DCL‐MgGa_2_Se_4_ single crystals.^[^ ^38^ ^]^ The measured results (Figure 3c) display that there is no obvious absorption peak in the range of 1.35–12.43 µm, indicating the IR transparent range that covers the two important IR atmospheric windows of 3–5 and 8–12 µm for the title compound, which is matched with the calculated IR spectrum (Figure S6, Supporting Information) and measured Raman spectrum (Figure S7, Supporting Information). Meanwhile, the Raman spectrum demonstrates the Mg—Se and Ga—Se chemical bonds in the structure of DCL‐MgGa_2_Se_4_. Specifically, the strong vibration peaks in the Raman spectrum at 79, 105, 129, and 178 cm^−1^ can be assigned to the stretching vibrations of the Mg—Se bonds in MgSe_4_ units, and the peaks at 222, 237, 250, and 304 cm^−1^ can be attributed to the characteristic vibrations of Ga—Se bonding in the GaSe_4_ groups.^[^ ^15^, ^28^, ^33^, ^39^ ^]^ What's more, the refractive index difference (RID) of DCL‐MgGa_2_Se_4_ was investigated by the polarizing microscope method.^[^ ^4^, ^10^, ^11^, ^40^ ^]^ The optical path differences of the DCL‐MgGa_2_Se_4_ crystal at 546 nm were 1.48 µm, and the thickness of the crystal was measured to be 31 µm (Figure S8, Supporting Information). According to the Equation (5) in the Experimental Section, the RID was calculated to be ≈0.048 at 546 nm, which is consistent with the PM behavior.

**Figure 4:**
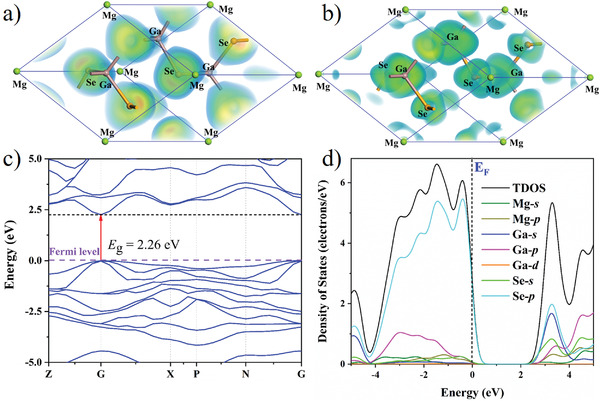
a) SHG‐density maps of the occupied and b) unoccupied orbitals in the VE process, c) calculated band gap, d) total density of states (TDOS) and partial density of states (PDOS) of DCL‐MgGa_2_Se_4_.

To detect the origin of optical properties in DCL‐MgGa_2_Se_4_, the first‐principles calculations based on density functional theory (DFT) were carried out.^[^ ^41^ ^]^ The calculated NLO coefficients of DCL‐MgGa_2_Se_4_ are −20.07 pm·V^−1^ for *d*_14_ and 4.05 pm·V^−1^ for *d*_15_, respectively. Through the SHG density method, it is found that in DCL‐MgGa_2_Se_4_, the occupied states are mainly from the contribution of nonbonding Se‐4*p* orbitals, while the unoccupied states are mainly the contribution of Ga‐4*s* 4*p*, Se‐4*p* (**Figure** **4**a,b). The calculated band structure (Figure 4c) shows that the title compound is a direct band gap compound. Considering the underestimation of the band gap in the standard DFT calculations with generalized gradient approximation (GGA), due to the discontinuity of the exchange‐correlation energy functional, the PBE0 calculation was also performed.^[^ ^42^ ^]^ Calculated PBE0 band gap of DCL‐MgGa_2_Se_4_ is 3.102 eV, which is in good agreement with the experimental result (2.96 eV). Moreover, the valence bands (VBs) at near the Fermi level is mainly occupied by Se *p* orbital with minor contribution of Mg *p* and Ga *p* orbitals, whereas the conduction bands (CBs) mainly originates from Se *s*, *p*, and Ga *s*, *p* orbitals in DCL‐MgGa_2_Se_4_ (Figure 4d), which is different from the MSe_4_ (M = Zn, Cd, and Hg) mainly determined band gaps in MGa_2_Se_4_ (M = Zn, Cd, and Hg) (Figure S9, Supporting Information).^[^ ^43^ ^]^ What's more, the natural bonding orbital (NBO) analysis indicates the HOMO‐LUMO gap of MgSe_4_ unit in DCL‐MgGa_2_Se_4_ is larger than that of ZnSe_4_ unit in ZnGa_2_Se_4_ (Figure S10, Supporting Information). The results indicate the positive contributions of AEM tetrahedron on the band gap in the AB_2_X_4_ family diamond‐like compounds.

## 3 Conclusion

In summary, the first DCL AEM‐contained selenide IR NLO material DCL‐MgGa_2_Se_4_ was designed and synthesized successfully. The results give a good example for the computer‐aided exploration of new IR NLO material. The developed DCL‐MgGa_2_Se_4_ exhibits a large experimental band gap (≈2.96 eV), high LIDT of ≈3.0 × AGS, wide transparency range (covering 3–12 µm), as well as a suitable SHG response of ≈0.9 × AGS with a type‐I PM behavior. Therefore, DCL‐MgGa_2_Se_4_ will be a promising IR NLO material. Meanwhile, DFT calculations indicate that the SHG response of DCL‐MgGa_2_Se_4_ is mainly originated from the GaSe_4_ and nonbonding Se‐4*p* orbitals, and demonstrate that AEM tetrahedral units can effectively broaden the band gap of CL compounds. These results enrich the diversity of CL compounds, and highlight the combination of calculation and experiment for the exploration of new IR NLO materials with excellent optical properties.

## 4 Experimental Section

### Structural Design and Prediction

The structure predictions were performed by the USPEX structure prediction method.^[^ ^29^ ^]^ The authors have been searching structures for 60 generations, and for the first generation, 120 structures were produced at random. Each subsequent generation produced 100 structures, in which 40% were produced by heredity, 20% were soft mutation, 20% were permutation operators, and 20% were produced using random symmetric and random topological generators.

For structural relaxation and thermodynamic properties calculation, the all‐electron projector augmented wave (PAW) method was implemented in the VASP code.^[^ ^44^ ^]^ The Perdew–Burke–Ernzerhof (PBE) of the generalized gradient approximation (GGA) was used to describe the exchange correlation potential.^[^ ^42a^ ^]^ During the calculations, plane‐wave kinetic energy cutoff of 600.0 eV and *Г*‐centered uniform k‐point meshes with reciprocal‐space resolution of 2*π* × 0.03 Å^−1^ were used.

### Property Calculations

Using the plane‐wave pseudopotential method implemented in the CASTEP, the electronic structure of MGa_2_Se_4_ (M = Mg, Zn, Cd, and Hg) and phonon spectra of MGSe‐I were performed on DFT.^[^ ^41^, ^42^ ^]^ The kinetic energy cut‐offs were set to be 990.0 eV with a density of fewer than 0.05 Å^−1^ in the Brillouin Zone (BZ) were adopted.^[^ ^45^ ^]^ Norm‐conserving pseudopotentials (NCP) were employed for each atomic species with the following valence configurations: Mg‐2*s* ^2^ 2*p* ^6^ 3*s* ^2^, Zn‐3*d* ^10^ 4*s* ^2^, Cd‐4*d* ^10^ 5*s* ^2^, Hg‐5*d* ^10^ 6*s* ^2^, Ga‐3*d* ^10^ *4*s^2^ 4*p* ^1^, and Se‐4*s* ^2^ 4*p* ^4^.^[^ ^45^ ^]^

SHG coefficients of the title compound were calculated with scissors operators revised. The so‐called length‐gauge formalism derived by Aversa and Sipe was adopted to calculate NLO properties.^[^ ^46^ ^]^ At a zero frequency, the static second‐order nonlinear susceptibilities could be described to virtual electrons) and virtual hole (VH) processes.

(1) $$\chi_{\alpha \beta \gamma }^{\left(2\right)}=\chi_{\alpha \beta \gamma }^{\left(2\right)}\left(\mathrm{VE}\right)+\chi_{\alpha \beta \gamma }^{\left(2\right)}\left(\mathrm{VH}\right)$$

where *χ*_*αβγ*_^(2)^(VE) and *χ*_*αβγ*_^(2)^(VH) are computed with the formulas as follows:

(2) $$\begin{array}{ccc} \chi_{\alpha \beta \gamma }^{\left(2\right)}\left(\mathrm{VE}\right) & = & \frac{e^{3}}{2\hbar^{2}m^{3}}\sum_{\nu cc^{\prime }}\int \frac{d^{3}k}{4\pi^{3}}P\left(\alpha \beta \gamma \right)Im\left[P_{c\nu }^{\alpha }P_{{\mathrm{cc}}^{\prime }}^{\beta }P_{c^{\prime }\nu }^{\gamma }\right]\\ & & \times \;\left(\frac{1}{\omega_{c\nu }^{3}\omega_{\nu c^{\prime }}^{2}}+\frac{2}{\omega_{\nu c}^{4}\omega_{c^{\prime }\upsilon }}\right) \end{array}$$

(3) $$\begin{array}{ccc} \chi_{\alpha \beta \gamma }^{\left(2\right)}\left(\mathrm{VH}\right) & = & \frac{e^{3}}{2\hbar^{2}m^{3}}\sum_{\nu \nu^{\prime }c}\int \frac{d^{2}k}{4\pi^{3}}P\left(\alpha \beta \gamma \right)\text{Im}\left[P_{\nu \nu^{\prime }}^{\alpha }P_{c\nu^{\prime }}^{\beta }P_{c\nu }^{\gamma }\right]\\ & & \times \;\left(\frac{1}{\omega_{c\nu }^{3}\omega_{\nu^{\prime }c}^{2}}+\frac{2}{\omega_{\nu c}^{4}\omega_{c\nu^{\prime }}}\right) \end{array}$$

here, *α*, *β*, *γ* are Cartesian components, and *v*/*v'*, *c*/*c'* denote VBs and CBs. And *P(αβγ)*, ℏ*ω_ij_*, and $p_{ij}^{\alpha }$ refer to full permutation, the band energy difference, and momentum matrix elements, respectively.

In this sum‐over‐states type formalism, the total SHG coefficient *χ* ^(2)^ was divided into contributions from VH and VE processes.

### Reagents and Syntheses

Mg powders (99.90%), Ga ingots (99.99%), and Se powders (99.99%) were purchased from Aladdin Biochemistry Technology Co., Ltd. and used without any further purification. MgSe was prepared by stoichiometric reaction of the Mg and Se powders at high temperature (≈950 °C).

DCL‐MgGa_2_Se_4_ was synthesized by high‐temperature solid state reactions. A mixture of MgSe, Ga, and Se in the molar ratio of 1:2:3 with a total mass of ≈0.7 g was weighed and loaded into a graphite crucible, then put it into a quartz tube and flame‐sealed with methane‐oxygen flame under a high vacuum of ≈10^−3^ Pa. The sealed tube was heated to 950 °C in 24 h, and kept at this temperature for 72 h in a computer‐controlled muffle furnace. After that, the furnace was cooled to 300 °C over 150 h, and then cooled to room temperature in 10 h. Finally, pare‐yellow powder samples were obtained.

### Structure Determination

Sub‐millimeter single crystals were selected under an optical microscope and used for the measurement of SXRD. The characterization was performed on a Bruker SMART APEX II CCD diffractometer using monochromatic Mo‐K*α* radiation (*λ* = 0.71073 Å) at room temperature. The data were corrected for Lorentz and polarization factors, and the absorption corrections were carried out by the SCALE program and integrated with the SAINT program.^[^ ^47^ ^]^ The direct methods and SHELXTL system were used to solve and refine the crystal structures.^[^ ^48^ ^]^ All of the atomic positions in the title compound were refined by full‐matrix least‐squares techniques. After refinement, the formula was determined to be MgGa_2_Se_4_. The final structure was checked for missing symmetry using the PLATON program, and no other higher symmetry elements were observed.^[^ ^49^ ^]^ Relevant crystallographic data, structural refinement details, atomic coordinates, equivalent isotropic displacement parameters, bond valence sum (BVS) calculation, anisotropic displacement parameters, and selected interatomic distances, angles are listed in Tables S1 and S3–S6, Supporting Information. CCDC 2124831 contains the supplementary crystallographic data for this paper. These data can be obtained free of charge from The Cambridge Crystallographic Data Centre via www.ccdc.cam.ac.uk/data_request/cif.

The elements analysis of the obtained DCL‐MgGa_2_Se_4_ single crystals was performed on a field emission scanning electron microscope (SEM) (JSM7610FPlus) equipped with an energy dispersive X‐ray spectrometer (Oxford X‐MaxN50), which was operated at 15 kV.

The purity of the synthesized MgGa_2_Se_4_ powder samples was confirmed by a Bruker D2 PHASER X‐ray diffractometer equipped with Cu K*α* radiation (*λ* = 1.5418 Å). The 2*θ* range was collected from 10° to 70°. The fixed counting time and scan step width were 1 s/step and 0.02°.

### UV‐Vis‐NIR Diffuse Reflectance Spectroscopy

The optical diffuse reflectance spectrum of DCL‐MgGa_2_Se_4_, ZnGa_2_Se_4_, CdGa_2_Se_4_, and HgGa_2_Se_4_ were measured by a Shimadzu SolidSpec‐3700DUV spectrophotometer in the wavelength range of 200–2600 nm at room temperature. The collected reflectance spectrum was transformed into the absorbance spectrum with the Kubelka–Munk function:

(4) $$\alpha /S={\left(1-R\right)}^{2}/2R$$

in which *α* is the absorption coefficient, *S* is the scattering coefficient, and *R* is the reflectance.^[^ ^36^ ^]^

### Optical Transmittance Spectrum

Single crystals of DCL‐MgGa_2_Se_4_ with a typical size of ≈180–212 µm were used to perform the measurements. The spectrum were collected with Hyperion microscope in Fourier transformer infrared system (Bruker Vertex 80v) that could focus the incident light to a small area of 50 × 50 µm, and CaF_2_ crystal plate well‐polished on both sides with sizes of 1 × 1 × 0.1 cm was used as transmitting medium.^[^ ^38^ ^]^ In addition, the scanning numbers and beam splitter were 128 and potassium bromide (KBr), respectively.

### Raman Spectroscopy

The Raman spectrum was measured on a LabRAM HR Evolution spectrometer equipped with a CCD detector using 532 nm radiation from a diode laser. Before the measurement, DCL‐MgGa_2_Se_4_ powder samples were put on a transparent glass slide, and an objective lens was used to choose a measured area on the crystal. The test was carried out at a maximum power of ≈60 mW with a spot size of ≈35 µm, and finished in 30 s.

### SHG Measurement

The SHG response of the title compound was evaluated with the Kurtz−Perry method on a Q‐switched laser (2090 nm, 50 ns, 3 Hz).^[^ ^35^ ^]^ AGS was used as the reference. For the measurements, DCL‐MgGa_2_Se_4_ and AGS samples were ground and sifted into different particle size ranges of 0–38, 38–55, 55–88, 88–105, 105–150, and 150–200 µm. The obtained samples were further poured into sample cells with a thickness of ≈1 mm. A photomultiplier tube and Tektronix oscilloscope were utilized to detect and recorded the frequency‐doubled intensity output from the samples.

### Refractive Index Difference Measurement

The RID of the title compound was estimated by using the polarizing microscope equipped (ZEISS Axio Scope. 5 pol) with Berek compensator.^[^ ^4^, ^10^, ^11^, ^40^ ^]^ The wavelength of the light source was 546 nm. The RID value is calculated by the following formula:

(5) $$R=\left|N_{g}-N_{p}\right|\times T=\Delta n\times T$$

in which *R* represents the optical path difference; *N*_g_, *N*_p_, and Δ*n* means the refractive index of fast light and slow light, the RID, respectively, and *T* denotes the thickness of the crystal.

### LIDT Measurement

The LIDT of the title compound was evaluated based on the ground micro‐crystals samples by a pulsed YAG laser (1.06 µm, 10 ns, 10 Hz). AGS powder samples with same particle size range were used as the reference. With the increase of laser energy, the color changes of the powder samples were constantly observed by optical microscope to determine the LIDT. It is worth noting that LIDT was related to the wavelength, pulse width, and spot size, and there was no accurate scaling law available. Therefore, the damage energy of DCL‐MgGa_2_Se_4_ and AGS were measured to be 0.15 and 0.05 mJ, respectively, at the same condition. The static LIDT behavior of title compound was estimated by comparing with AGS.

(6) $$\begin{array}{ccc} L_{DCL-MgGa_{2}Se_{4}} & = & L_{AGS}\left(\;\frac{\frac{E_{\mathrm{DCL}-MgGa_{2}Se_{4}}}{\pi r^{2}t}}{\frac{E_{AGS}}{\pi r^{2}t}}\right)\\ & = & L_{AGS}\left(\;\frac{E_{\mathrm{DCL}-MgGa_{2}Se_{4}}}{E_{AGS}}\right)\;\approx 3L_{AGS} \end{array}$$

where *L* denotes LIDT, *E* is the energy of a single pulse, *r* is the spot radius, and *t* is pulse width.

### Statistical Analyses

The statistical analyses on the ternary DCL AEM selenide, the coordination numbers of M atoms, and the bond lengths of M—Se and in MSe*_n_* polyhedra (M = Be, Mg, Ca, Sr, and Ba; *n* ≥ 4) (Figure S1a, Supporting Information) were carried out on basis of the data in ICSD (ICSD ‐ 4.7.0, the latest release of ICSD ‐ 2021/10/25). The statistic results on the band gaps of all experimentally verified PM selenide IR NLO materials (without cationic co‐occupation, and with *E*_g_ ≥ 2.0 eV) in Figure 3d and Table S7, Supporting Information, were implemented on basis of the published papers and books.

## Conflict of Interest

The authors declare no conflict of interest.

## Supporting information

Supporting Information

## Data Availability

The data that support the findings of this study are available from the corresponding author upon reasonable request.
