# Supplementary material for: The Combination of Structure Prediction and Experiment for the Exploration of Alkali‐Earth Metal‐Contained Chalcopyrite‐Like IR Nonlinear Optical Material
Source: Adv Sci (Weinh). 2022 Apr 11;9(15):2106120. doi: 10.1002/advs.202106120 (PMC9130896; doi:10.1002/advs.202106120)
Supplement: Supplementary file 1 — Supporting Information [file ADVS-9-2106120-s001.pdf]

Supporting Information

The Combination of Structure Prediction and Experiment for the Exploration of Alkali-Earth Metal-Contained Chalcopyrite-Like IR Nonlinear Optical Material

*Peng Wang, Yu Chu, Abudukadi Tudi, Congwei Xie, Zhihua Yang, Junjie Li** *and Shilie Pan**

Contents

Table S1. The coordination numbers of Mg in known Mg-based ternary selenides without cationic co-occupation.

Table S2. Crystallographic information and PBE0 band gap of predicted MGSe (I-VI).

Table S3. Atomic coordinates, isotropic displacement parameters and BVS calculation for DCL-$MgGa_2Se_4$.

Table S4. Anisotropic displacement parameters (Å^2 × 10^3) for DCL-$MgGa_2Se_4$.

Table S5. Bond Lengths for DCL-$MgGa_2Se_4$.

Table S6. Bond Angles for DCL-$MgGa_2Se_4$.

Table S7. Summary of experimentally verified PM selenide IR NLO materials (without cationic co-occupation) with $E_g \geq 2.0$ eV.

Figure S1. (a) Statistics on bond length and the coordination of AEM-based selenides without cationic co-occupation. (b) The crystal stucture of the known Be-based selenide BeSe ($F\bar{4}3m$).

Figure S2. The experimental and theoretical PXRD patterns of DCL-$MgGa_2Se_4$.

Figure S3. EDS spectrum showing the existence of Mg, Ga and Se elements in the micrometer DCL-$MgGa_2Se_4$ crystal. Insets showing the corresponding SEM image, EDS mapping and qualified analyses of EDS spectrum.

Figure S4. The comparison of optical properties between DCL-$MgGa_2Se_4$ and the benchmark IR NLO materials showing a good balance of band gap and SHG response in DCL-$MgGa_2Se_4$.

Figure S5. UV-Vis-NIR diffuse-reflectance spectra and transformed solid-state optical absorption spectra of $ZnGa_2Se_4$ (a), $CdGa_2Se_4$ (b) and $HgGa_2Se_4$ (c) powder samples.

Figure S6. The calculated IR vibrational spectrum of DCL-$MgGa_2Se_4$.

Figure S7. The Raman spectrum of DCL-$MgGa_2Se_4$.

Figure S8. (a) The crystal size measured by using a Bruker SMART APEX II CCD diffractometer, (b) the interference colors observed in the cross-polarized light of the crystal selected for the refractive index difference (RID) measurement of DCL-$MgGa_2Se_4$.

Figure S9. (a) Calculated band gap, (b) total density of states (TDOS) and partial density of states (PDOS) of $ZnGa_2Se_4$; (c) Calculated band gap, (d) TDOS and PDOS of $CdGa_2Se_4$; (e) Calculated band gap, (f) TDOS and PDOS of $HgGa_2Se_4$.

Figure S10. The schematic molecular orbital energy level diagram of the [$MgSe_4$] unit in DCL-$MgGa_2Se_4$ (a) and [$ZnSe_4$] unit in $ZnGa_2Se_4$ (b).

Table S1. The coordination numbers of Mg in known Mg-based ternary selenides without cationic co-occupation.[a]

Compounds	Space Group	Coordination of Mg	ICSD Number	CL?
MgSe	$Fm\bar{3}m$	$MgSe_6$	53946	×
$MgSe_2$	$Ia\bar{3}$	$MgSe_6$	642815	×
MgSe	$P6_3mc$	$MgSe_4$	658986	×
$Mg_2Al_2Se_5$	$P\bar{3}m1$	$MgSe_6$	41928	×
Mg_2GeSe_4	$Pnma$	$MgSe_6$	252050	×
$Mg_2P_2Se_6$	$R\bar{3}$	$MgSe_6$	413165	×
$MgLu_2Se_4$	$Fd\bar{3}m$	$MgSe_4$	44912	×
$MgTm_2Se_4$	$Fd\bar{3}m$	$MgSe_4$	76051	×
MgY_2Se_4	$Fd\bar{3}m$	$MgSe_4$	76052	×
$MgYb_2Se_4$	$Fd\bar{3}m$	$MgSe_4$	76053	×
$MgAl_2Se_4$	$R\bar{3}m$	$MgSe_6$	83363	×
$MgTm_2Se_4$	$Fd\bar{3}m$	$MgSe_4$	198603	×
$MgYb_2Se_4$	$Fd\bar{3}m$	$MgSe_4$	198604	×
MgU_8Se_{17}	$C2/m$	$MgSe_6$	601593	×
$MgEr_2Se_4$	$Fd\bar{3}m$	$MgSe_4$	630754	×
$MgHo_2Se_4$	$Fd\bar{3}m$	$MgSe_4$	639387	×
$MgSc_2Se_4$	$Fd\bar{3}m$	$MgSe_4$	642814	×
Mg_2SiSe_4	$Pnma$	$MgSe_6$	642818	×
$Mg2SnSe_4$	$Pnma$	$MgSe_6$	642819	×
CS-$MgGa_2Se_4$ [1]	$R\bar{3}m$	$MgSe_6$	-	×
**DCL-$MgGa_2Se_4$**	**$I\bar{4}$**	**$MgSe_4$**	**This work**	√
$Cs_2MgSn_3Se_8$	$P2_12_12_1$	$MgSe_4$	193778	×
$Cs_2MgGe_3Se_8$	$P2_12_12_1$	$MgSe_4$	193784	×
$Na_4MgGe_2Se_6$	$C2$	$MgSe_6$	239112	×
$Na_4MgSi_2Se_6$	$C2$	$MgSe_6$	239113	×
$K_2MgP_2Se_6$	$P2/n$	$MgSe_6$	413168	×
$Cu_2MgSiSe_4$	$Pmn2_1$	$MgSe_4$	425556	×

[a] There are no Be-based ternary selenides in the ICSD (ICSD - 4.7.0, the latest release of ICSD - 2021/10/25).

Table S2. Crystallographic information and PBE0 band gap of predicted MGSe (I-VI).

	Space Group	Z	Cell Parameter	V (Å^3)	$E_{g,\ PBE0}$ (eV)
MGSe-I	$I\bar{4}$	2	$a = b = 5.81440$ Å, $c = 10.96900$ Å, $\alpha = \beta = \gamma = 90°$	370.831717	3.10
MGSe-II	$R\bar{3}m$	3	$a = b = 3.95370$ Å, $c = 41.25820$ Å, $\alpha = \beta = 90°$, $\gamma = 120°$	558.532392	1.57
MGSe-III	$P\bar{3}m1$	1	$a = b = 3.95420$ Å, $c = 13.62220$ Å, $\alpha = \beta = 90°$, $\gamma = 120°$	184.457006	1.55
MGSe-IV	$C2/m$	2	$a = 6.84946$ Å, $b = 3.95268$ Å, $c = 13.87358$ Å, $\alpha = \gamma = 90°$, $\beta = 91.9626°$	375.389152	2.37
MGSe-V	$I\bar{4}2m$	2	$a = b = 5.55280$ Å, $c = 11.89520$ Å, $\alpha = \beta = \gamma = 90°$	366.771711	1.58
MGSe-VI	$P\bar{4}2m$	1	$a = b = 5.55630$ Å, $c = 5.95320$ Å, $\alpha = \beta = \gamma = 90°$	183.789993	2.45

Table S3. Atomic coordinates, isotropic displacement parameters and BVS calculation for DCL-$MgGa_2Se_4$.

**Atoms**	**Wyckoff Position**	**Occupancy**	***x***	***y***	***z***	**U(eq)**	**BVS**
Mg1	2a	1	5000	5000	5000	17(1)	2.063
Ga1	2d	1	10000	5000	7500	16(1)	2.947
Ga2	2b	1	10000	0	5000	13(1)	2.995
Se1	8g	1	7547(2)	2306(1)	6355(1)	14(1)	2.001

Table S4. Anisotropic displacement parameters ($Å^2 \times 10^3$) for DCL-$MgGa_2Se_4$. The anisotropic displacement factor exponent takes the following form:
$-2\pi^2[h^2a^{*2}U_{11}+\cdots+2hka^*b^*U_{12}]$.

Atom	U_{11}	U_{22}	U_{33}	U_{23}	U_{13}	U_{12}
Mg1	15(2)	15(2)	21(3)	0	0	0
Ga1	18(1)	18(1)	12(1)	0	0	0
Ga2	14(1)	14(1)	13(1)	0	0	0
Se1	14(1)	14(1)	14(1)	-1(1)	0(1)	1(1)

Table S5. Bond Lengths for DCL-$MgGa_2Se_4$.

Atom	Atom	Length/Å	Atom	Atom	Length/Å
Mg1	Se1	2.5647(8)	Ga1	Se1[2]	2.4125(7)
Ga1	Se1	2.4125(7)	Ga1	Se1[3]	2.4125(7)
Ga1	Se1[1]	2.4125(7)	Ga2	Se1	2.4074(8)

Symmetry transformations used to generate equivalent atoms:

[1] -x+2, -y+1, z; [2] -y+3/2, x-1/2, -z+3/2; [3] y+1/2, -x+3/2, -z+3/2

Table S6. Bond Angles for DCL-$MgGa_2Se_4$.

Atom	Atom	Atom	Angle/°	Atom	Atom	Atom	Angle/°
Se1	Mg1	$Se1^{7}$	108.738(14)	$Se1^{1}$	Ga1	$Se1^{3}$	105.017(12)
$Se1^{7}$	Mg1	$Se1^{8}$	108.738(14)	$Se1^{2}$	Ga1	$Se1^{3}$	118.80(3)
Se1	Mg1	$Se1^{8}$	110.95(3)	$Se1^{1}$	Ga1	Se1	118.80(3)
Se1	Mg1	$Se1^{9}$	108.737(14)	Se1	Ga2	$Se1^{4}$	111.382(16)
$Se1^{9}$	Mg1	$Se1^{8}$	108.738(14)	Se1	Ga2	$Se1^{5}$	105.71(3)
$Se1^{7}$	Mg1	$Se1^{9}$	110.95(3)	$Se1^{4}$	Ga2	$Se1^{5}$	111.382(16)
$Se1^{1}$	Ga1	$Se1^{2}$	105.017(12)	$Se1^{5}$	Ga2	$Se1^{6}$	111.382(16)
$Se1^{3}$	Ga1	Se1	105.017(12)	$Se1^{4}$	Ga2	$Se1^{6}$	105.71(3)
$Se1^{2}$	Ga1	Se1	105.017(12)	Se1	Ga2	$Se1^{6}$	111.381(16)

Symmetry transformations used to generate equivalent atoms:

^{1} -x+2, -y+1, z; ^{2} -y+3/2, x-1/2, -z+3/2; ^{3} y+1/2, -x+3/2, -z+3/2; ^{4} -y+1, x-1, -z+1;

^{5} -x+2, -y, z; ^{6} y+1, -x+1, -z+1; ^{7} y, -x+1, -z+1; ^{8} -x+1, -y+1, z; ^{9} -y+1, x, -z+1

Table S7. Summary of experimentally verified PM selenide IR NLO materials (without cationic co-occupation) with $E_g \geq 2.0$ eV

Compound	Space Group	$E_{g, exp.}$ (eV)	Ref.
$LiGaSe_2$	$Pna2_1$	3.57	[2]
DCL-$MgGa_2Se_4$	$I\bar{4}$	2.96	This work.
$LiInSe_2$	$Pna2_1$	2.86	[3]
$Na_4MgSi_2Se_6$	$C2$	2.85	[4]
β-$BaGa_4Se_7$	$Pna2_1$	2.82	[5]
α-$BaGa_4Se_7$	Pc	2.64	[6]
$SnGa_4Se_7$	Pc	2.55	[7]
$Na_4MgGe_2Se_6$	$C2$	2.53	[4]
$Li_2ZnGeSe_4$	Pn	2.5	[8]
$Li_2CdGeSe_4$	$Pna2_1$	2.5	[9]
$BaZnGeSe_4$	$Ama2$	2.5	[10]
$BaHgGeSe_4$	$Ama2$	2.49	[11]
β-$BaGa_2Se_4$	$I4cm$	2.49	[12]
$Li_2BaSiSe_4$	$I\bar{4}2m$	2.47	[13]
$SrHgGeSe_4$	Ama2	2.42	[11]
$Li_2BaGeSe_4$	$I\bar{4}2m$	2.4	[14]
$Na_2Ge_2Se_5$	$Pna2_1$	2.38	[15]
$CsZrPSe_6$	$Pmc2_1$	2.3	[16]
$KAg_3Ga_8Se_{14}$	Cm	2.27	[17]
$EuCdGeSe_4$	$Ama2$	2.25	[18]
$Li_2CdSnSe_4$	$Pna2_1$	2.25	[9]
$RbPSe_6$	$Pca2_1$	2.18	[19]
$Li_2BaSnSe_4$	$I\bar{4}2m$	2.18	[14]
$NaIn_3Se_5$	$P3_2$	2.17	[20]
$KPSe_6$	$Pca2_1$	2.16	[19]
$Cs_4Ge_4Se_{12}$	$Pna2_1$	2.15	[21]
$AgInSe_2$	$I\bar{4}2d$	2.14	[22]
δ-Ga_2Se_3	$C222_1$, $P\bar{4}$ and $C222$	2.10	[23]
$Rb_4Ge_4Se_{12}$	$Pna2_1$	2.10	[21]
$K_2P_2Se_6$	$Pna2_1$	2.08	[24]
$SrHgSnSe_4$	$Ama2$	2.07	[25]

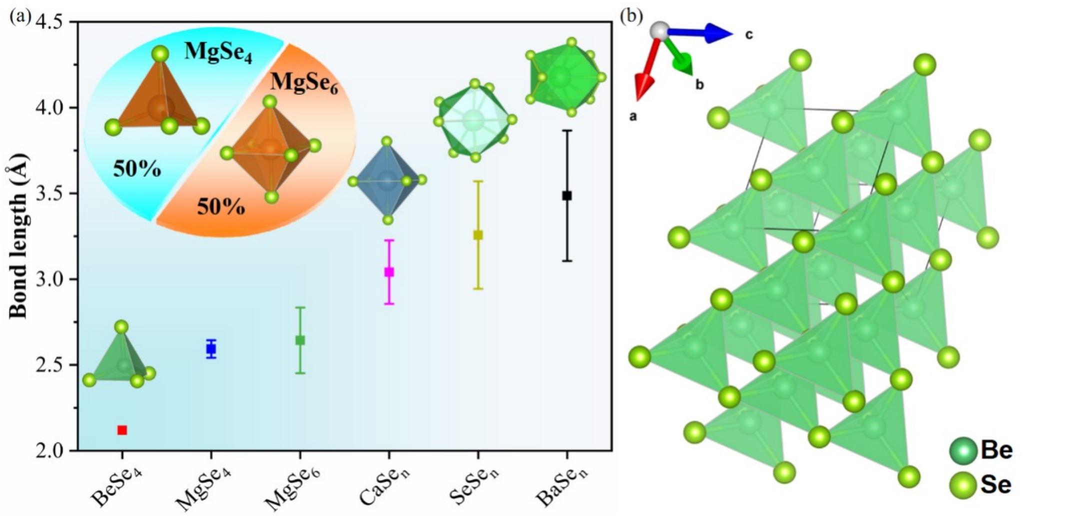

Figure S1. (a) Statistics on bond length and the coordination of AEM-based selenides without cationic co-occupation. (b) The crystal stucture of the known Be-based selenide BeSe ($F\bar{4}3m$).

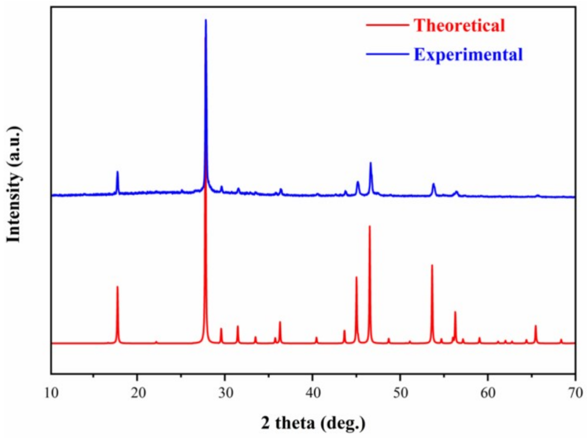

Figure S2. The experimental and theoretical PXRD patterns of DCL-$MgGa_2Se_4$.

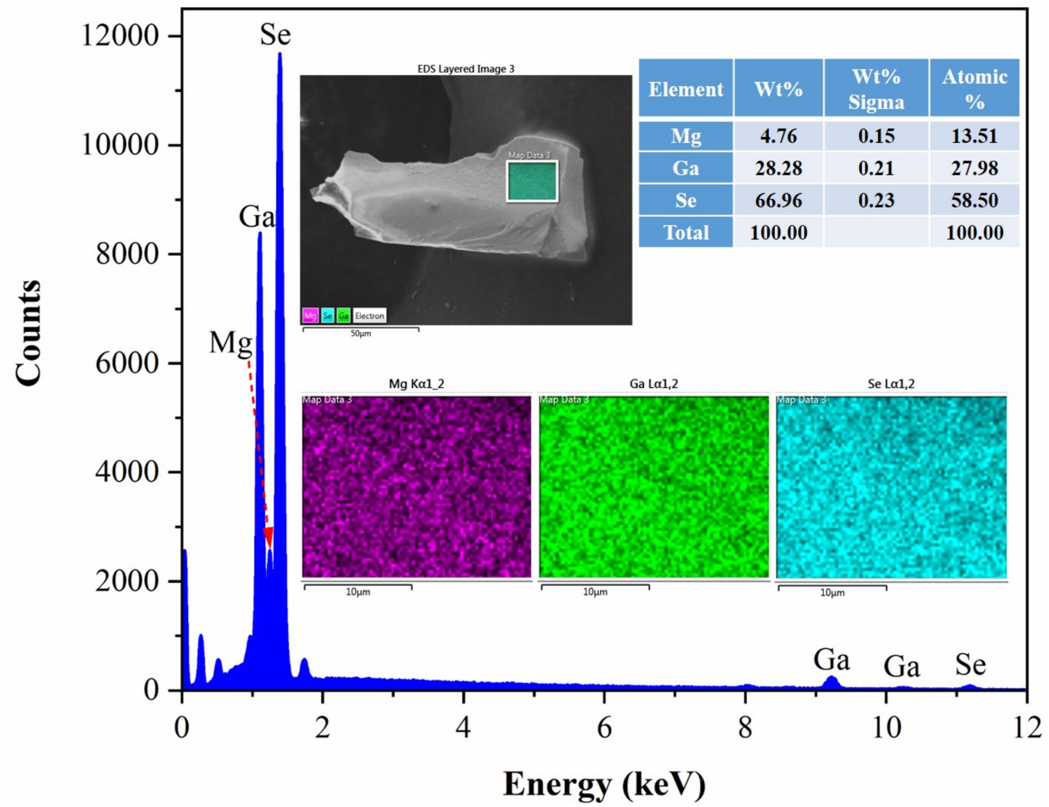

Figure S3. EDS spectrum showing the existence of Mg, Ga and Se elements in the micrometer DCL-$MgGa_2Se_4$ crystal. Insets showing the corresponding SEM image, EDS mapping and qualified analyses of EDS spectrum.

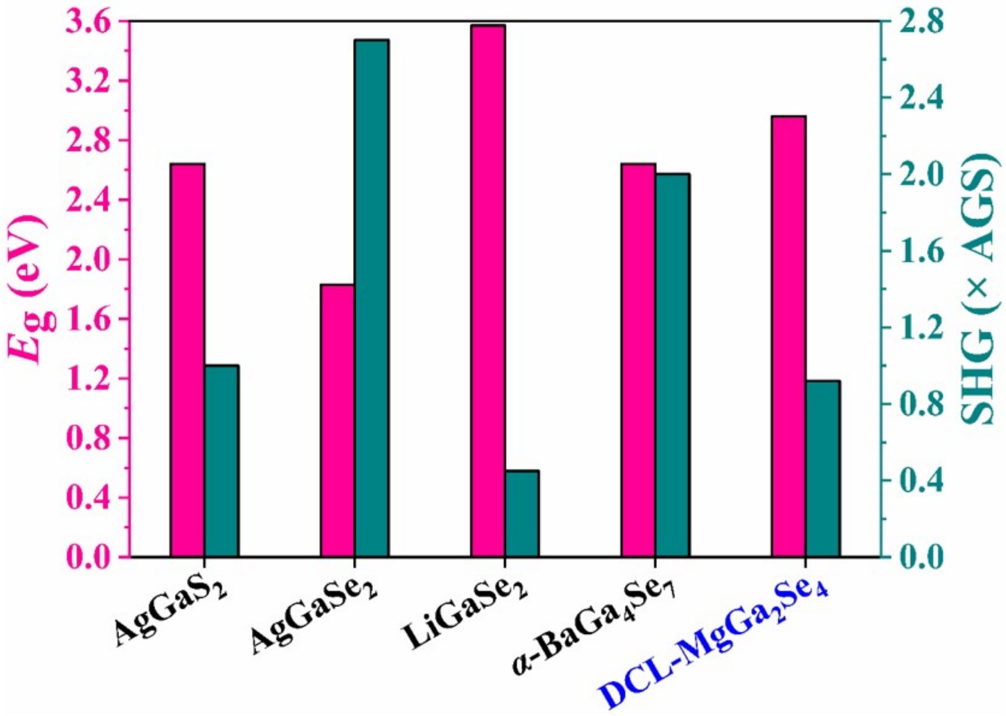

Figure S4. The comparison of optical properties between DCL-$MgGa_2Se_4$ and the benchmark IR NLO materials showing a good balance of band gap and SHG response in DCL-$MgGa_2Se_4$.

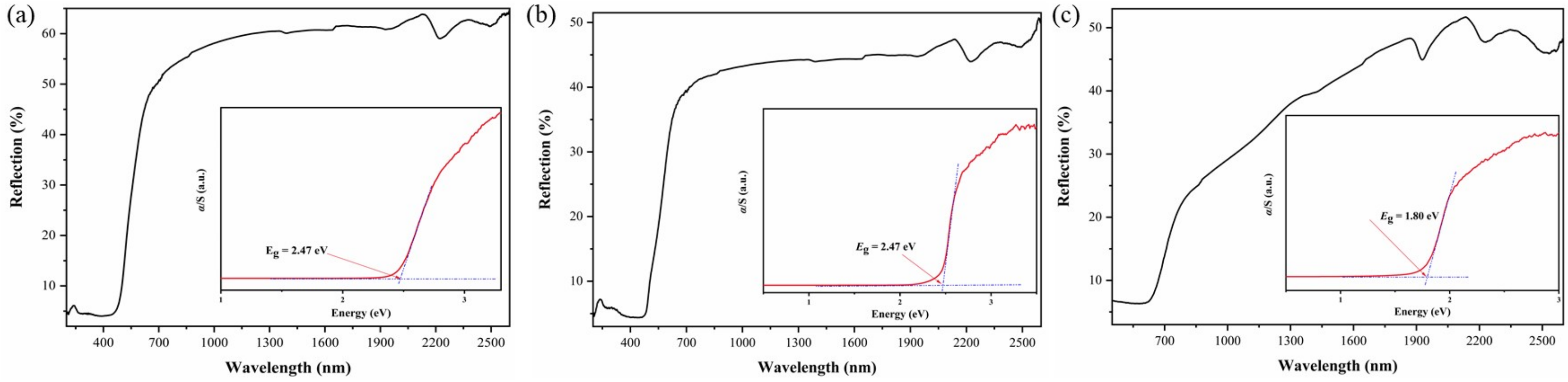

Figure S5. UV-Vis-NIR diffuse-reflectance spectra and transformed solid-state optical absorption spectra of $ZnGa_2Se_4$ (a), $CdGa_2Se_4$ (b) and $HgGa_2Se_4$ (c) powder samples.

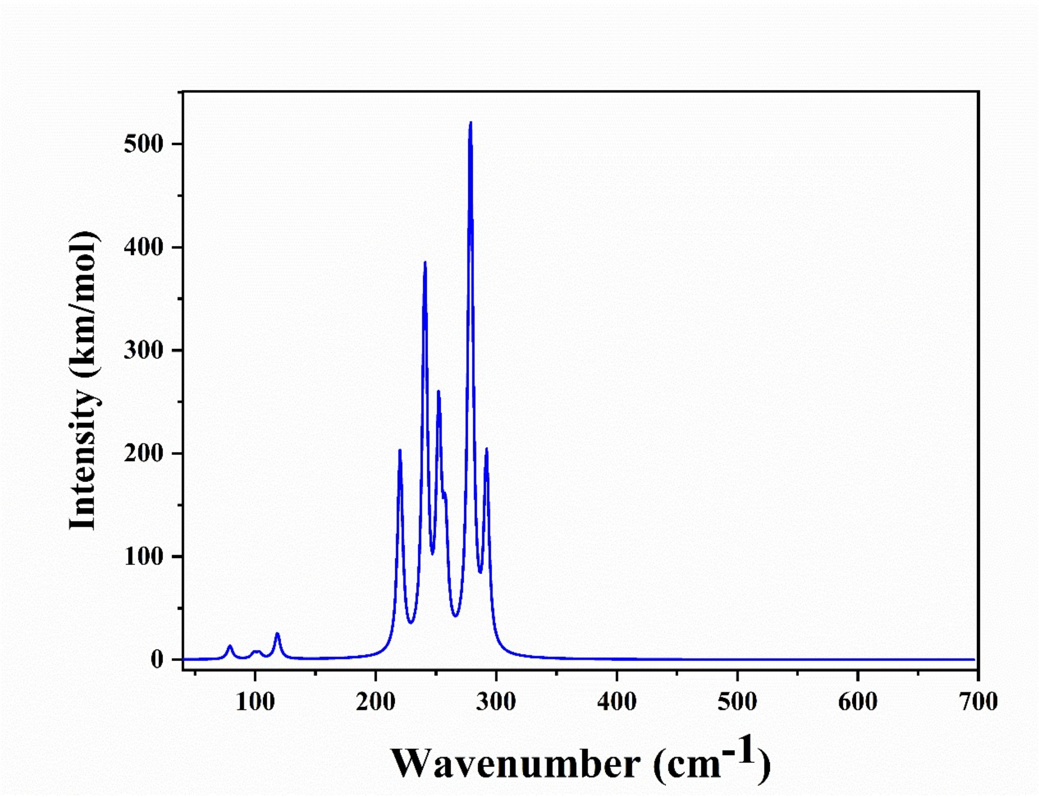

Figure S6. The calculated IR vibrational spectrum of DCL-$MgGa_2Se_4$.

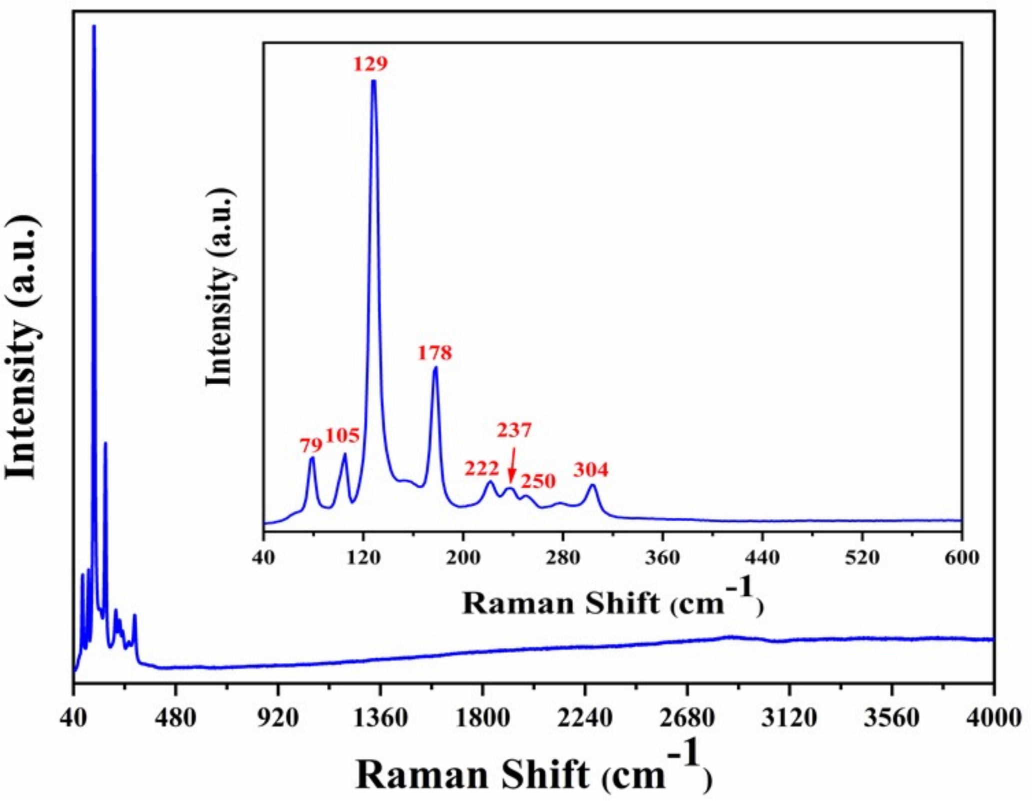

Figure S7. The Raman spectrum of DCL-$MgGa_2Se_4$ (the inset is the local diagram of Raman spectrum with wavenumber range of 40 - 600 cm^{-1}).

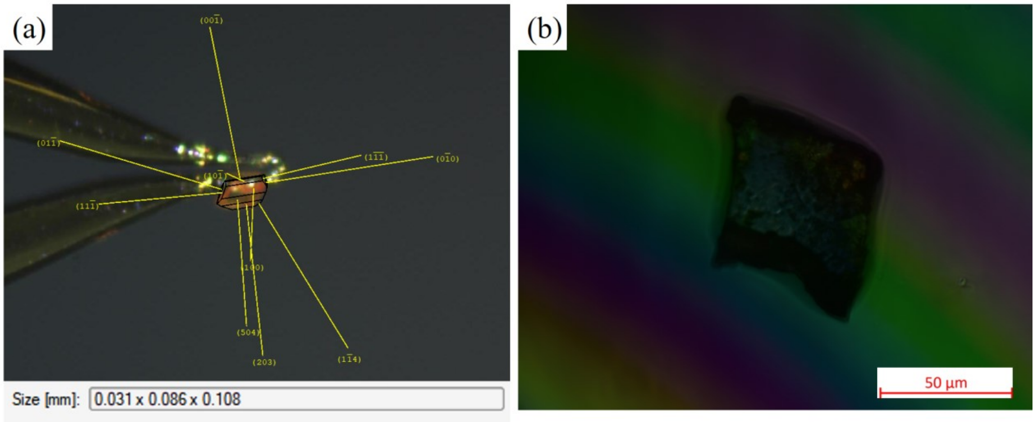

Figure S8. (a) The crystal size measured by using a Bruker SMART APEX II CCD diffractometer, (b) the interference colors observed in the cross-polarized light of the crystal selected for the refractive index difference (RID) measurement of DCL-$MgGa_2Se_4$.

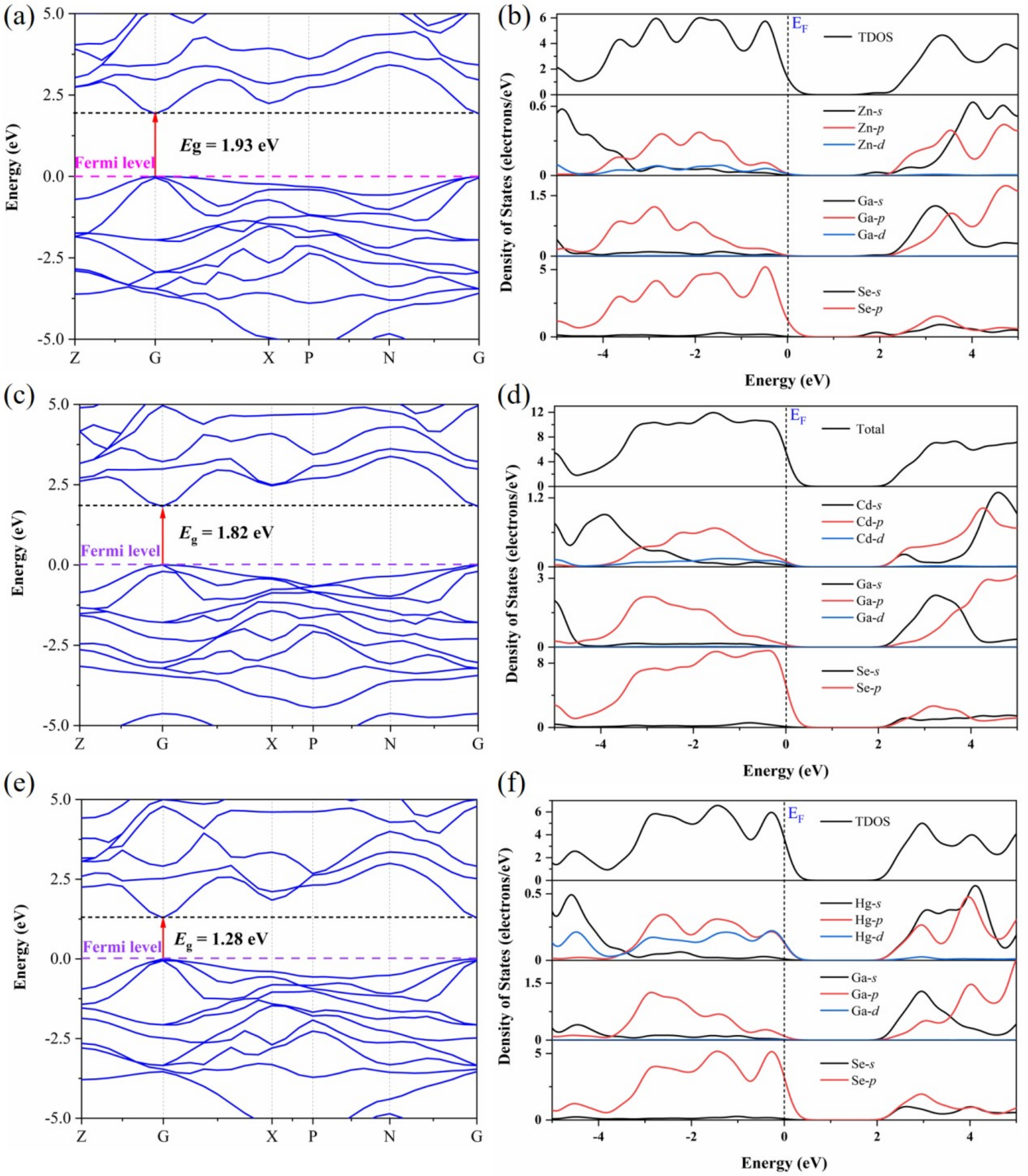

Figure S9. (a) Calculated band gap, (b) total density of states (TDOS) and partial density of states (PDOS) of $ZnGa_2Se_4$; (c) Calculated band gap, (d) TDOS and PDOS of $CdGa_2Se_4$; (e) Calculated band gap, (f) TDOS and PDOS of $HgGa_2Se_4$.

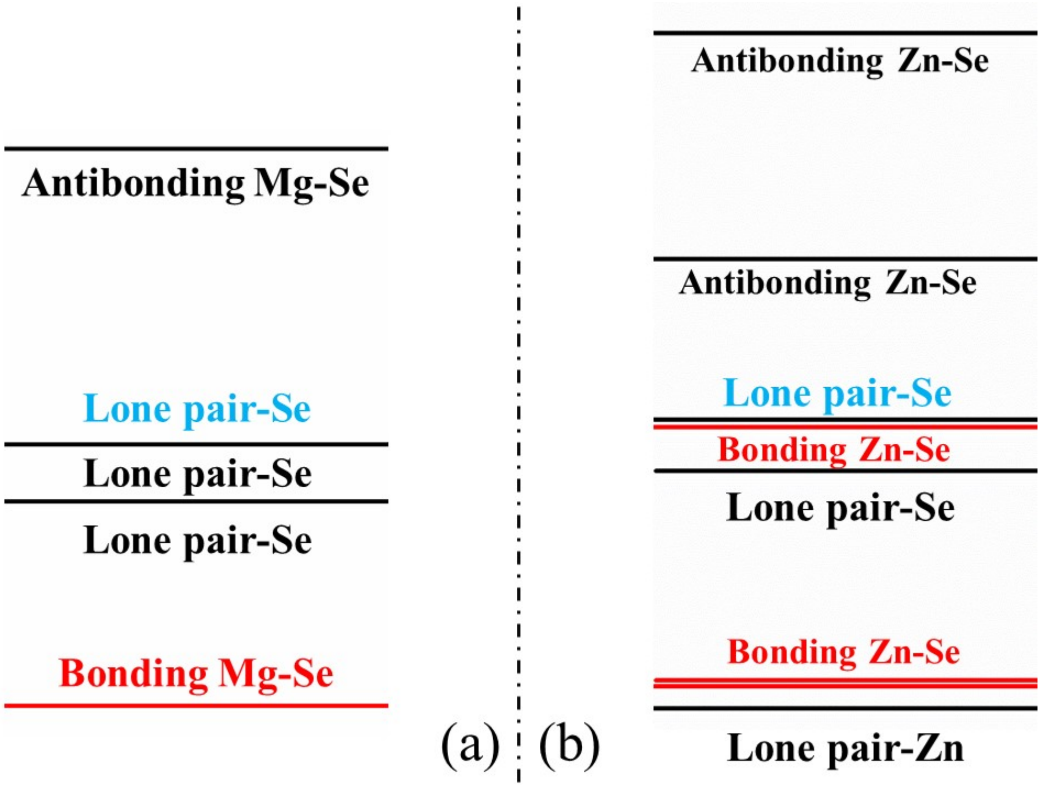

Figure S10. The schematic molecular orbital energy level diagram of the [$MgSe_4$] unit in DCL-$MgGa_2Se_4$ (a) and [$ZnSe_4$] unit in $ZnGa_2Se_4$ (b).

References

[1] a) H. G. Kim, W. T. Kim, Y. G. Kim, *Phys. Rev. B* **1988**, *38*, 9469; b) H. G. Kim, W. T. Kim, Y. G. Kim, *Phys. Rev. B* **1989**, *39*, 10429; c) H. G. Kim, W. T. Kim, Y. g. Kim, *Phys. Rev. B* **1989**, *40*, 1329; d) *$MgGa_2Se_4$ Crystal Structure: Datasheet from "PAULING FILE Multinaries Edition - 2012" in SpringerMaterials (https://materials.springer.com/isp/crystallographic/docs/sd_0314153)*, Springer-Verlag Berlin Heidelberg & Material Phases Data System (MPDS), Switzerland & National Institute for Materials Science (NIMS), Japan.

[2] D. N. Nikogosyan, *Nonlinear Optical Crystals: A Complete Survey*, Springer-Verlag, New York, **2005**.

[3] A. Yelisseyev, F. Liang, L. Isaenko, S. Lobanov, A. Goloshumova, Z. S. Lin, *Opt. Mater.* **2017**, *72*, 795.

[4] K. Wu, Z. H. Yang, S. L. Pan, *Inorg. Chem.* **2015**, *54*, 10108.

[5] Z. Qian, Q. Bian, H. P. Wu, H. W. Yu, Z. S. Lin, Z. G. Hu, J. Y. Wang, Y. C. Wu, *J. Mater. Chem. C* **2021**, *1*, 3777.

[6] J. Y. Yao, D. J. Mei, L. Bai, Z. S. Lin, W. L. Yin, P. Z. Fu, Y. C. Wu, *Inorg. Chem.* **2010**, *49*, 9212.

[7] W. D. Cheng, C. S. Lin, H. Zhang, Y. Z. Huang, G. L. Chai, *ChemPhysChem* **2017**, *18*, 519.

[8] a) J. H. Zhang, D. J. Clark, J. A. Brant, C. W. Sinagra, Y. S. Kim, J. I. Jang, J. A. Aitken, *Dalton Trans.* **2015**, *44*, 11212; b) A. Weiland, J. H. Zhang, D. J. Clark, J. A. Brant, C. W. Sinagra, Y. S. Kim, J. I. Jang, J. A. Aitken, *Dalton Trans.* **2017**, *46*, 10102.

[9] J. H. Zhang, D. J. Clark, A. Weiland, S. S. Stoyko, Y. S. Kim, J. I. Jang, J. A. Aitken, *Inorg. Chem. Front.* **2017**, *4*, 1472.

[10] W. L. Yin, A. K. Iyer, C. Li, J. Y. Yao, A. Mar, *J. Alloys Compd.* **2017**, *708*, 414.

[11] Y. W. Guo, F. Liang, W. L. Yin, Z. Li, X. Y. Luo, Z. S. Lin, J. Y. Yao, A. Mar, Y. C. Wu, *Chem. Mater.* **2019**, *31*, 3034.

[12] Y. J. Zhang, Q. Bian, H. P. Wu, H. W. Yu, Z. G. Hu, J. Y. Wang, Y. C. Wu, *Angew. Chem. Int. Ed.* **2021**, *60*, 2.

[13] G. M. Li, K. Wu, S. L. Pan, *Chin. Sci. Bull.* **2019**, *64*, 1671.

[14] K. Wu, B. B. Zhang, Z. H. Yang, S. L. Pan, *J. Am. Chem. Soc.* **2017**, *139*, 14885.

[15] I. Chung, J. H. Song, J. I. Jang, A. J. Freeman, M. G. Kanatzidis, *J. Solid State Chem.* **2012**, *195*, 161.

[16] S. Banerjee, C. D. Malliakas, J. I. Jang, J. B. Ketterson, M. G. Kanatzidis, *J. Am. Chem.*

Soc. **2008**, *130*, 12270.

[17] J. N. Li, W. D. Yao, X. H. Li, W. L. Liu, H. G. Xue, S. P. Guo, *Chem. Commun.* **2021**, *57*, 1109.

[18] W. h. Xing, N. Z. Wang, Y. W. Guo, Z. Li, J. Tang, K. J. Kang, W. L. Yin, Z. S. Lin, J. Y. Yao, B. Kang, *Dalton Trans.* **2019**, *48*, 17620.

[19] a) I. Chung, J. Do, C. G. Canlas, D. P. Weliky, M. G. Kanatzidis, *Inorg. Chem.* **2004**, *43*, 2762; b) I. Chung, J. I. Jang, C. D. Malliakas, J. B. Ketterson, M. G. Kanatzidis, *J. Am. Chem. Soc.* **2009**, *132*, 384.

[20] S. F. Li, X. M. Jiang, B. W. Liu, D. Yan, C. S. Lin, H. Y. Zeng, G. C. Guo, *Chem. Mater.* **2017**, *29*, 1796.

[21] B. W. Liu, M. Y. Zhang, X. M. Jiang, S. F. Li, H. Y. Zeng, G. Q. Wang, Y. H. Fan, Y. F. Su, C. S. Li, G. C. Guo, J. S. Huang, *Chem. Mater.* **2017**, *29*, 9200.

[22] L. Isaenko, P. Krinitsin, V. Vedenyapin, A. Yelisseyev, A. Merkulov, J. J. Zondy, V. Petrov, *Cryst. Growth Des.* **2005**, *5*, 1325.

[23] S. P. Guo, X. Y. Cheng, Z. D. Sun, Y. Chi, B. W. Liu, X. M. Jiang, S. F. Li, H. G. Xue, S. Q. Deng, V. Duppel, J. Köhler, G. C. Guo, *Angew. Chem. Int. Ed.* **2019**, *58*, 8087.

[24] I. Chung, C. D. Malliakas, J. I. Jang, C. G. Canlas, D. P. Weliky, M. G. Kanatzidis, *J. Am. Chem. Soc.* **2007**, *129*, 14996.

[25] Y. W. Guo, F. Liang, Z. Li, W. H. Xing, Z. S. Lin, J. Y. Yao, A. Mar, Y. C. Wu, *Inorg. Chem.* **2019**, *58*, 10390.
